# Economic analyses of venous thromboembolism prevention strategies in hospitalized patients: a systematic review

**DOI:** 10.1186/cc11241

**Published:** 2012-03-09

**Authors:** Subarna Thirugnanam, Ruxandra Pinto, Deborah J Cook, William H Geerts, Robert A Fowler

**Affiliations:** 1 Department of Medicine, University of Toronto, Toronto, Ontario, Canada; 2 Department of Critical Care Medicine, Sunnybrook Health Sciences Centre, University of Toronto, Toronto, Ontario, Canada; 3 Department of Medicine, St. Joseph's Hospital and McMaster University, Hamilton, Ontario, Canada; 4 Department of Medicine, Sunnybrook Health Sciences Centre, University of Toronto, Toronto, Ontario, Canada

## Abstract

**Introduction:**

Despite evidence-based guidelines for venous thromboembolism prevention, substantial variability is found in practice. Many economic evaluations of new drugs for thromboembolism prevention do not occur prospectively with efficacy studies and are sponsored by the manufacturers, raising the possibility of bias. We performed a systematic review of economic analyses of venous thromboembolism prevention in hospitalized patients to inform clinicians and policy makers about cost-effectiveness and the potential influence of sponsorship.

**Methods:**

We searched MEDLINE, EMBASE, Cochrane Databases, ACP Journal Club, and Database of Abstracts of Reviews of Effects, from 1946 to September 2011. We extracted data on study characteristics, quality, costs, and efficacy.

**Results:**

From 5,180 identified studies, 39 met eligibility and quality criteria. Each addressed pharmacologic prevention: low-molecular-weight heparins versus placebo (five), unfractionated heparin (12), warfarin (eight), one or another agents (five); fondaparinux versus enoxaparin (11); and rivaroxaban and dabigatran versus enoxaparin (two). Low-molecular-weight heparins were most economically attractive among most medical and surgical patients, whereas fondaparinux was favored for orthopedic patients. Fondaparinux was associated with increased bleeding events. Newer agents rivaroxaban and dabigatran may offer additional value. Of all economic evaluations, 64% were supported by manufacturers of a "new" agent. The new agent had a favorable outcome in 38 (97.4%) of 39 evaluations [95% confidence interval [CI] (86.5 to 99.9)]. Among studies supported by a pharmaceutical company, the sponsored medication was economically attractive in 24 (96.0%) of 25 [95% CI, 80.0 to 99.9)]. We could not detect a consistent bias in outcome based on sponsorship; however, only a minority of studies were unsponsored.

**Conclusion:**

Low-molecular-weight heparins and fondaparinux are the most economically attractive drugs for venous thromboembolism prevention in hospitalized patients. Approximately two thirds of evaluations were supported by the manufacturer of the new agent; such drugs were likely to be reported as economically favorable.

## Introduction

Venous thromboembolism occurs in up to 40% of hospitalized medical and surgical patients in the absence of prophylactic anticoagulation [1,2]. Even with prophylaxis, the risk of venous thromboembolism in critically ill patients approaches 10% and has serious consequences: untreated pulmonary embolism has a mortality rate approaching 25% [3-5]. Among critically ill patients, those developing venous thromboembolism have longer intensive care unit and hospital stays, longer duration of mechanical ventilation, and higher hospital mortality [6]. Consequently, venous thromboembolism not only is associated with serious morbidity and mortality, but also has major implications for healthcare resource utilization.

Appropriate use of prophylaxis to prevent venous thromboembolism in patients at risk has been identified as one of the most important patient-safety interventions for hospitals [7]. However, substantial variability is found in the use of such prophylaxis in practice. Prevention is most commonly achieved with anticoagulant drugs. Because important decisions about pharmacologic interventions are made with knowledge of their economic consequences, formal economic analyses are useful tools to guide clinicians and policy makers about the value of drug interventions and their consequences [8,9]. However, many evaluations of new drugs do not occur prospectively with efficacy studies, and many are sponsored by the manufacturers, raising the possibility of bias.

We performed a systematic review of economic analyses of venous thromboembolism-prevention strategies in acutely ill hospitalized patients. Our objectives were to review and critically appraise the economic evaluations of a broad spectrum of strategies in diverse patient groups to help inform clinicians and policy makers about the cost-effectiveness of various approaches to venous thromboembolism prophylaxis.

## Materials and methods

### Date sources and searches

We searched MEDLINE, EMBASE, the Cochrane Database of Systematic Reviews, ACP Journal Club, Database of Abstracts of Reviews of Effects (DARE), and Cochrane Controlled Trials Register from 1946 to October 21, 2011, by using a combination of the following subject headings and text words: *venous thrombosis, pulmonary embolism, low-molecular-weight heparin, LMWH, dalteparin, enoxaparin, nadroparin, tinzaparin, heparin, unfractionated heparin, UFH, anticoagulants, warfarin, aspirin, fondaparinux, rivaroxaban, dabigatran, intermittent pneumatic compression devices, compression stockings, vena cava filters, venous foot pump, economics, health care cost, cost-effectiveness analysis, cost-benefit analysis*, and *economic analysis *(Additional file 1). No limits regarding publication type were initially applied. To identify additional potentially relevant studies, we checked the reference lists of identified systematic and narrative reviews and the personal files of the authors and collaborators. We also sent the full list of identified articles and inclusion criteria to venous thromboembolism experts in the field to identify additional published or relevant unpublished studies.

### Study selection

**Figure 1:**
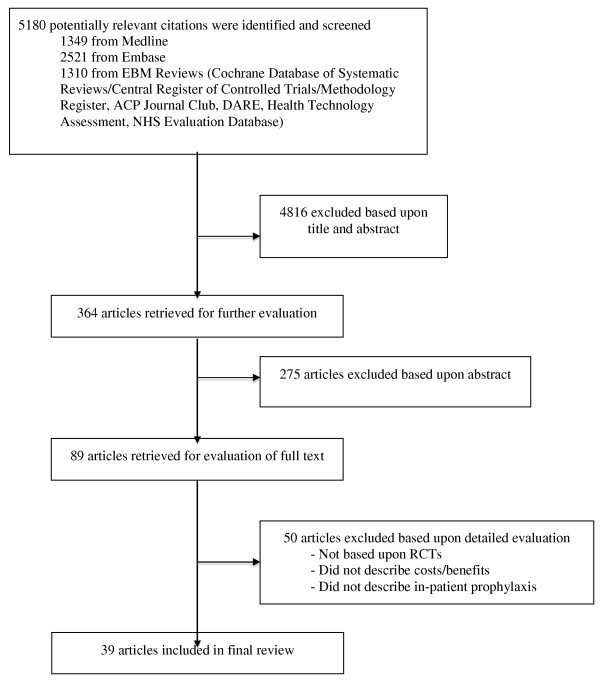
**Study eligibility diagram**.

From 5,180 potentially relevant citations, 4,816 were excluded based on title and abstract review (Figure 1). The full text versions of 89 manuscripts were retrieved for full evaluation. Two reviewers (ST, RF) independently assessed each of the articles and applied the following eligibility criteria: (a) the economic evaluation was based on data from randomized controlled trials or meta-analyses of randomized controlled trials; (b). the study described hospitalized patients; (c) the study compared at least two different venous thromboembolism prophylaxis strategies; (d) the study described drug-acquisition costs, the costs of providing prophylaxis, costs of complications (including venous thromboembolism treatment and prophylaxis failures); and (e) the study described the effect of prophylaxis with respect to the number of venous thromboembolism events prevented and diagnosed. We excluded evaluations based on the following study designs: 1. cohort studies or other observational studies; 2. studies on outpatient use of venous thromboembolism prophylaxis; 3. studies on the treatment of venous thromboembolism; 4. studies examining the efficacy of short-term versus long-term venous thromboembolism prophylaxis; 5. decision analytic models based on data from nonrandomized trials; 6. studies examining anticoagulants for conditions other than venous thromboembolism, and seven letters, editorials, or narrative reviews of economic issues in venous thromboembolism prophylaxis. We also excluded studies appraised as low to moderate quality, as defined later.

### Data extraction and quality assessment

We critically appraised each article by using established criteria [9]. Our goal was to include only those studies that adhered to a high methodologic quality. We assigned an ordinal score of quality based on the criteria set forth in the "User's guide to the medical literature: XIII. How to use an article on economic analysis of clinical practice" [9]. With a semiquantitative scale incorporating these characteristics, we assigned 1 point for each of the 12 categories. All studies were graded as high (≥ 9 points), medium (5 to 8 points), or low (0 to 4 points) quality by two authors (ST, RF). Disagreements about the inclusion of individual studies were resolved by consensus between two authors (ST, RF). Of the 84 articles selected for full review, 50 were appraised as low or moderate quality, and the remaining 39 studies were selected for data abstraction.

We identified 10 economic evaluations of mechanical prophylaxis, including intermittent pneumatic compression devices, compression stockings, or vena cava filters [10-17]. None of these articles met our previously stated eligibility criteria.

From each included study, we abstracted the following: the patient group, venous thromboembolism prophylaxis strategy, duration of prophylaxis, time frame of the study, source of the outcome data, source of the cost data, incremental costs and benefits of each strategy, results of any sensitivity analyses, the country in which the study was performed, and the declared source of funding for the economic analysis. We attempted to contact authors of studies for which no external support was declared to ensure that this was the case.

We abstracted the number of thrombotic events, costs, and complication rates of the prophylaxis and of the treatment of venous thromboembolism from each article. We then recorded or calculated the incremental cost-efficacy ratio for each venous thromboembolism prophylaxis strategy. If we were unable to calculate the incremental cost-efficacy ratio because of missing data, we attempted to contact the authors to obtain this ratio or original data. Costs were converted to 2009 US dollars and adjusted for country-specific temporal changes in gross domestic product [18-20]. We standardized the incremental effects we reported as "venous thromboembolism events avoided," "life-years or quality adjusted life-years gained' or 'deaths avoided per 1000 patients', as is commonly performed in venous thromboembolism literature. We chose in-hospital or near-term (< 90 days) events for the primary comparisons whenever possible, as the short and longer-term effects of inpatient thromboprophylaxis are often greatest during this time period.

### Data synthesis and analysis

Heterogeneity of the interventions, perspectives, and time-horizons precluded meta-analytic techniques to combine incremental cost-efficacy ratios into a single summary statistic. We summarized cost-effectiveness ratios by graphic representation of point estimates on a cost-efficacy plane. Categoric variables and proportions were compared by using the χ^2 ^or Exact tests as appropriate.

## Results

### Study comparisons, populations, and format

Among the 39 studies included in this review, the following comparisons were made: low-molecular-weight heparins versus placebo (five) [20-24]; unfractionated heparin versus low-molecular-weight heparins (12) [21,23-33]; various low-molecular-weight heparins versus warfarin (eight) [34-41]; various low-molecular-weight heparins compared with one another or other agents (five) [31,42-45]; fondaparinux versus enoxaparin (11) [46-56], rivaroxaban versus low-molecular-weight heparins or dabigatran [57], and dabigatran versus low-molecular-weight heparin [58] (Tables 1 and 2).

**Table 1:** Study characteristics

Study characteristics	Number of studies
**Thromboprophylaxis compared**	
Low-molecular-weight heparin versus placebo	5
Low-molecular-weight heparin versus unfractionated heparin	12
Low-molecular-weight heparin versus warfarin	8
Low-molecular-weight heparin versus fondaparinux	11
Other	7
**Patient population**	
Orthopedic surgery	26
Other surgical	5
Medical	8
**Funding**	
Industry	25
Other or unknown	14
**Geographic perspective**	
US	18
UK	6
Continental Europe	11
Canada	4

**Table 2:** Study description and quality assessment

Article	Interventions compared	Patient group	Were the outcomes accurately measured?	Were the costs accurately measured?	Was uncertainty in analysis determined?	Were estimates and costs related to the baseline risk in treatment population?
Pechevis, 2000	Enoxaparin 40 mg daily versus placebo for 6-14 days	Medical	Yes; outcomes taken from RCTs	Yes; data from literature	Yes	Yes
Lloyd, 2001	UFH 5,000 units twice daily versus enoxaparin 40 mg daily for 6-14 days	Medical	Yes; outcomes taken from RCTs	Yes; data from literature	Yes	Yes
Lamy, 2002	Enoxaparin 20 mg versus 40 mg versus placebo for 6-14 days	Medical	Yes; outcomes taken from RCTs	Yes; data from hospital, OHIP	Yes	Yes
Offord, 2003	Enoxaparin 40 mg daily versus UFH 5,000 units twice daily versus none for 6-14 days	Medical	Yes; outcomes taken from RCT/meta-analysis	Yes; data from a hospital	Yes	Yes
Schadlich, 2006	Enoxaparin 40 mg versus UFH 5,000 units three times daily for 6-14 days	Medical	Yes; outcomes taken from RCTs/meta-analysis	Yes; data from the German Health System	Yes	Yes
Drummond, 1994	UFH 5,000 units 3 times daily versus enoxaparin 40 mg daily for 7 days	HFS	Yes; outcomes taken from RCTs	Yes; data from literature	Yes	Yes
Hawkins, 1997	Enoxaparin 30 mg daily versus UFH 5,000 units for 7 days	THR	Yes; outcomes taken from RCTs	Yes; data from literature	Yes	Yes
Marchetti, 1999	UFH 5,000 units twice daily versus LMWH enoxaparin 40 mg daily for 14 days	THR	Yes; outcomes taken from RCTs/meta-analysis	Yes; data from literature	Yes	Yes
McGarry, 2004	UFH 5,000 units twice daily versus enoxaparin 40 mg daily versus nothing for 30 days	Medical	Yes; outcomes taken from RCTs/meta-analysis	Yes; data from literature	Yes	Yes
Deitelzweig, 2008	UFH 5,000 units twice daily versus enoxaparin 40 mg daily versus nothing for 5 days	Medical	Yes; outcomes taken from RCTs	Yes; data from literature	Yes	Yes
Wade, 2008	UFH 5,000 units 3 times daily versus dalteparin 5,000 units daily for 10 days	Gynecology oncology surgery	Yes; outcomes taken from RCTs	Yes; data from literature	Yes	Yes
Lloyd, 1997	UFH 5,000 units twice daily/3 times daily versus nadroparin for 10-14 days	Orthopedic and general surgery	Yes; outcomes taken from meta-analysis	Yes; data from published rates of pay, costs from a hospital	Yes	Yes
Heerey, 2005	Dalteparin 2,500 units versus 5,000 units versus UFH for 10 days	Abdominal surgery	Yes; outcomes taken from RCTs	Yes; data from Medicare reimbursement	Yes	Yes
O'Brien, 1994	Enoxaparin 30 mg twice daily versus warfarin for 7 days	THR	Yes; outcomes taken from RCTs	Yes; data from literature	Yes	Yes
Menzin, 1995	Enoxaparin 30 mg twice daily versus warfarin (INR 2-3) versus nothing for 5-14 days	THR	Yes; outcomes taken from RCTs	Yes; data from literature	Yes	Yes
Hull, 1997	Warfarin versus tinzaparin 175 units/kg for 14 days	THR, TKR	Yes; outcomes taken from RCTs	Yes; data from literature	Yes	Yes
Hawkins, 1998	Enoxaparin 30 mg twice daily versus warfarin for 10 days	TKR	Yes; outcomes taken from RCTs	Yes; data from literature	Yes	Yes
Francis, 1999	Dalteparin 2,500 units, then 5,000 units versus warfarin for 10 days	THR	Yes; outcomes taken from RCTs	Yes; costs from participating hospitals in RCT	Yes; for costs	Yes
Botteman, 2002	Enoxaparin 30 mg daily versus warfarin 5 mg daily for 7 days	THR	Yes; outcomes taken from RCTs	Yes; data from literature	Yes	Yes
Caprini, 2002	Enoxaparin 30 mg twice daily for 7 days versus UFH 5,000 units 3 times daily and warfarin for 10 days	THR	Yes; outcomes taken from RCTs	Yes; data from literature	Yes	Yes
Levin, 2001	Desirudin 15 mg twice daily versus enoxaparin 40 mg daily for 10 days	THR	Yes; outcomes taken from RCTs	Yes; data from literature	Yes	Yes
Honorato, 2004	Bemiparin 3,500 units daily versus enoxaparin 40 mg daily for 8-12 days	TKR	Yes; outcomes taken from RCTs	Yes; data from National Health Care Institute, pharmacists association	Yes	Yes
Attanasio, 2001	Dermatan sulfate 300 mg daily versus UFH 5,000 units 3 times daily for 7 days	Surgical oncology	Yes; outcomes taken from RCTs	Yes - data from hospital costs	Yes	Yes
Wade, 2001	Tinzaparin 3,500 units versus enoxaparin 30 mg twice daily for 8 weeks	Spinal cord injury	Yes; outcomes taken from RCTs	Yes; data from different hospitals, DRG	Yes	Yes
						**Were estimates and costs related to the baseline risk in treatment population- are these results generalizable?**
Gordois, 2003	Enoxaparin 40 mg daily versus fondaparinux 2.5 mg daily for 5-9 days	THR, HFS	Yes; outcomes taken from RCTs	Yes; data from NICE	Yes	Yes
Lundkvist, 2003	Fondaparinux 2.5 mg daily versus enoxaparin 40 mg daily for 7 days	THR, HFS	Yes; outcomes taken from RCTs	Yes; data from literature	Yes	Yes
Wade, 2003	Fondaparinux 2.5 mg daily versus enoxaparin 40 mg daily versus 30 mg twice daily for 7-10 days	HFS	Yes; outcomes taken from RCTs	Yes; data from literature	Yes	Yes
Szucs, 2003	Fondaparinux 2.5 mg daily versus enoxaparin 40 mg daily for 7 days	THR, TKR HFS	Yes; outcomes taken from RCTs	Yes; data from literature and surveys in Switzerland	Yes	Yes
Sullivan, 2004	Fondaparinux 2.5 mg daily versus enoxaparin 40 mg daily for 7 days	THR, TKR HFS	Yes; outcomes taken from RCTs	Yes; costs from review of 220 acute care hospitals	Yes	Yes
Dranitsaris, 2004	Fondaparinux 2.5 mg daily versus enoxaparin 40 mg daily for 7 days	THR, HFS	Yes; outcomes taken from a meta-analysis	Data from CIHI, surveys	Yes	Yes
Spruill, 2004	Fondaparinux 2.5 mg daily versus enoxaparin 30 mg twice daily for 4-5 days	TKA	Yes; outcomes taken from RCTs	Yes; data from literature	Yes	Yes
Spruill, 2004	Fondaparinux 2.5 mg daily versus enoxaparin 30 mg twice daily for 10 days	THR	Yes; outcomes taken from RCTs	Yes; data from literature	Yes	Yes
Wade, 2004	Fondaparinux 2.5 mg daily versus enoxaparin 40 mg daily for 7 days	HFS	Yes; outcomes taken from RCTs	Yes; data from literature	Yes	Yes
Bjorvatn, 2005	Fondaparinux 2.5 mg daily versus enoxaparin 40 mg daily for 7 days	THR, TKR HFS	Yes; outcomes taken from RCTs	Yes; data from Norwegian national sources	Yes	Yes
Wolowacz 2009	THR Dabigatran 220 mg daily versus enoxaparin 40 mg daily for 28-35 days TKR Dabigatran 220 mg daily for versus enoxaparin 40 mg daily 6-10 days	THR, TKR	Yes; outcomes taken from RCTs	Yes; data from UK national sources	Yes	Yes
McCullagh, 2009	THR Dabigatran 220 mg daily for 35 days versus rivaroxaban 10 mg daily for 35 days versus enoxaparin 40 mg daily for 14 days TKR Dabigatran 220 mg daily for 14 days versus rivaroxaban 10 mg daily for 10 days versus enoxaparin 40 mg daily for 10 days	THR, TKR	Yes; outcomes taken from RCTs	Yes; data from literature and Irish national sources	Yes	Yes
Pechevis, 2000	Yes	N/R	No	Yes	Yes	Yes
Lloyd, 2001	Yes	N/R	No	Yes	Yes	Yes
Lamy, 2002	Yes	N/R	No	Yes	Yes	Yes
Offord, 2003	Yes	N/R	No	Yes	Yes	Yes
Schadlich, 2006	Incompletely	N/R	No	Yes	Yes	Yes
Drummond, 1994	Incompletely	N/R	No	Yes	Yes	Likely
Hawkins, 1997	Yes	N/R	No	Yes	Yes	Yes
Marchetti, 1999	Incompletely	N/R	No	Yes	Yes	Yes
Etchells, 1999	Yes	N/R	No	Yes	Yes	Yes
McGarry, 2004	Incompletely	N/R	No	Yes	Yes	Yes
**Article**	**Were incremental costs and outcomes measured?**	**Do incremental costs and outcomes differ between subgroups?**	**Does allowance for uncertainty change results?**	**Are prophylaxis benefits worth the harm and costs?**	**Generalizability: could other patient populations expect similar outcomes?**	**Generalizability: could other patient populations expect to experience similar costs?**
Heerey, 2005	Incompletely	N/R	No	Yes	Yes	Likely
Deitelzweig, 2008	Yes	N/R	No	Yes	Yes	Likely
Wade, 2008	Yes	Yes	Yes	Yes	Yes	Yes
O'Brien, 1994	Yes	N/R	No	Yes	Yes	Yes
Menzin, 1995	Yes	N/R	No	Yes	Yes	Yes
Hull, 1997	Yes	N/R	No	Yes	Yes	Yes
Hawkins, 1998	Yes	N/R	No	Yes	Yes	Yes
Francis, 1999	Yes	Yes	Yes	Likely	Yes	Yes
Botteman, 2002	Incompletely	N/R	No	Yes	Perhaps	Yes
Nerurkar, 2002	Incompletely	N/R	No	Yes	Perhaps	Yes
Levin, 2001	Incompletely	N/R	No	Yes	Yes	Likely
Caprini, 2002	Yes	Yes	No	Yes	Yes	Likely
	**Were incremental costs and outcomes measured?**	**Do incremental costs and outcomes differ between subgroups?**	**Does allowance for uncertainty change results?**	**Are prophylaxis benefits worth the harm and costs?**	**Generalizability: could other patient populations expect similar outcomes?**	**Generalizability: could other patient populations expect to experience similar costs?**
Honorato, 2004	Yes	N/R	No	Yes	Yes	Yes
Wade, 2001	Incompletely	N/R	No	Yes	Yes	Yes
Gordois, 2003	Yes	N/R	No	Yes	Yes	Yes
Wade, 2003	Yes	N/R	No	Yes	Yes	Yes
Annemans, 2004	Yes	N/R	No	Yes	Yes	Yes
Attanasio, 2001	Yes	N/R	No	Yes	Yes	Yes
Szucs, 2003	Yes	Yes	No	Yes	Yes	Yes
Sullivan, 2004	Yes	Yes	No	Yes	Yes	Yes
Dranitsaris, 2004	Yes	N/R	No	Yes	Yes	Yes
Spruill, 2004	Yes	N/R	No	Yes	Yes	Yes
Spruill, 2004	Yes	N/R	No	Yes	Yes	Yes
Wade, 2004	Yes	N/R	No	Yes	Yes	Yes
Bjorvatn, 2005	Yes	Yes	No	Yes	Yes	Yes
Wolowacz 2009	Yes	Yes	No	Yes	Yes	Yes
McCullagh 2009	Yes	Yes	No	Yes	Yes	Yes

CIHI, Canadian Institute for Health Information; DRG, diagnosis-related group; HFS, hip fracture surgery; NICE, National Centre for Clinical Excellence

N/R, not reported; OHIP, Ontario Health Insurance Plan; RCT, randomized controlled trial; THR, total hip replacement; TKR, total knee replacement; UFH, unfractionated heparin.

Twenty-six evaluations were performed in orthopedic patients [25-27,34-43,46-58]; five in other surgical populations [28,30-32,44], and eight in medical patients [20-24,29,33,45]. All 39 studies were either decision analytic models based on individual randomized controlled trials [20-22,24,26,28,31-49,51-58] or meta-analysis [23,27,29,30,51].

### Study perspectives, time horizon, and funding

The studies were conducted from a North American or European economic perspective: 18 of the studies were conducted in the United States [26,29,31-41,45,48,51-53,55], six in the United Kingdom [21,23,25,46,57,58], four in Canada [23,28,34,51], and three in Italy [27,30,44], two in Sweden [42,47], and one each in Spain [43], Belgium [49], France [20], Norway [54], Switzerland [56], and Germany [24]. Seven studies were conducted from the perspective of the hospital [24,41,42,44,45,49,53], four from a societal perspective [20,28,30,32], nine from the perspective of another specific payer [23,31,33,40,54-58], and the remaining 19 studies did not clearly specify which perspective was used.

The prophylaxis time horizons considered were variable: either for 5 days [33,51]; 7 days [25,28,34,38,44,46-50,53-56]; 6 to 14 days [23]; 10 days [31,32,37,40,41,52], 8 to 12 days [43]; 7 to 14 days [26]; 6 to 14 days [57], 10 to 14 days [20,22-24,27,30,35,36,39,42-46]; 30 days [29], 8 to 33 days [59], and another for 8 weeks [45]. Twenty-five studies received some sponsorship by pharmaceutical companies [20,22-25,28,30,33-38,40,41,43,44,46,47,50,54-56,58].

### Study quality

All 39 studies that were of *high *quality were included in this systematic review (Table 2). Eighteen of these showed complete cost data but did not present complete effectiveness data [25-27,29,31,37-45,51,54-56]. The remaining 17 studies had complete cost and effectiveness data. Six of the studies obtained effectiveness data from meta-analysis [23,25,27,31,50,57], whereas the remainder obtained effectiveness data from one or more randomized controlled trials. Six of these studies obtained effectiveness data from a single trial [20,22-24,33,41]. All studies, with the exception of one [23], obtained cost data from multiple sources, including actual and estimated healthcare system costs, randomized controlled trials, literature reviews, and other national government sources. All performed sensitivity analyses of some description.

### Cost and effect estimates

#### Low Molecular Weight Heparins versus Placebo

Among the five studies comparing low-molecular-weight heparins with placebo in medical patients, enoxaparin was the most economically attractive strategy in all five studies and dominant in two [20,22], with incremental cost-efficacy ratios ranging from $83 to $1,711 per venous thromboembolism event avoided in three others [21,23,24]; cost per life-year or quality-adjusted life-year gained were not investigated (Table 3). Sensitivity analysis did not alter these findings. Four of the five studies were sponsored by the manufacturer of enoxaparin [20-22,24].

**Table 3:** Incremental costs, effects, and cost-efficacy ratios for the different modes of venous thromboembolism prophylaxis

Low-molecular-weight heparins versus placebo						
Reference	Patient population	Incremental cost (USD)	Incremental effects (VTE avoided or life-years or QALYS gained)	ICER (USD/VTE event avoided or life-years or QALYS gained)	Bleeding complications	Most economically attractive drug
*Pechevis, 2000	Medical	Net saving (value not reported) per 1,000 patients with enoxaparin	94 DVT/PE avoided, four lives (estimated 12 life-years) gained, per 1,000 patients with enoxaparin	Enoxaparin dominant	Not reported	Enoxaparin
Lloyd, 2001	Medical	$20,680 per 1,000 patients with enoxaparin	20 VTE events avoided per 1,000 patients with enoxaparin	$1, 034 per VTE avoided with enoxaparin	Six more major bleeding events per 1,000 patients with enoxaparin	Enoxaparin
*Lamy, 2002	Medical	$1, 910 per 1,000 patients in tertiary care setting with enoxaparin	2.3% fewer VTE events with enoxaparin	$83 per VTE avoided with enoxaparin	Not reported	Enoxaparin
*Offord, 2004	Medical	Net saving ($26,478) per 1,000 patients with enoxaparin	14 VTE events and 3.5 deaths avoided per 1,000 patients with enoxaparin	Enoxaparin dominant	Not reported	Enoxaparin
*Schaldich, 2006	Medical	$44,665 per 1,000 patients with enoxaparin	26 VTE events avoided per 1,000 patients with enoxaparin	$1, 711 per VTE avoided with enoxaparin	Not reported	Enoxaparin
**Low-molecular-weight heparins versus unfractionated heparin**						
**Reference**	**Patient population**	**Incremental cost (USD)**	**Incremental effects (VTE avoided or life-years or QALYS gained)**	**ICER (USD/VTE event avoided or life-years or QALYS gained)**	**Bleeding complications**	**Most economically attractive drug**
*Drummond, 1994, enoxaparin	HFS	Net saving ($43,609) per 1,000 patients with enoxaparin	Four deaths avoided per 1,000 patients with enoxaparin	Enoxaparin dominant	Not reported	Enoxaparin
*Hawkins, 1997, enoxaparin	THR	$57,972 per 1,000 patients with enoxaparin	47 DVT events avoided per 1,000 patients with enoxaparin	$1, 180 per VTE event avoided with enoxaparin	Not reported (implied enoxaparin increased bleeding risk)	Enoxaparin
Marchetti, 1999, enoxaparin	THR	Net saving ($90,000) per 1,000 patients with enoxaparin	70 life-years gained per, 1000 patients with enoxaparin	Enoxaparin dominant	Not reported	Enoxaparin
*Etchells, 1999, enoxaparin	Colorectal surgery	$180,641 per 1,000 patients with enoxaparin	0 VTE events avoided with enoxaparin	UFH dominant	12 additional major bleeding events with enoxaparin	UFH
Lloyd, 2001, enoxaparin	Medical	Net saving ($850) per 1,000 patients with enoxaparin	21 VTE events avoided per 1,000 patients with enoxaparin	Enoxaparin dominant	18 fewer major bleeding events with Enoxaparin	Enoxaparin
*Offord, 2003, enoxaparin	Medical	Net saving ($54,649) per 1,000 patients with Enoxaparin	20.5 VTE events and 0.5 deaths avoided per 1,000 patients with enoxaparin	Enoxaparin dominant	Not reported	Enoxaparin
*McGarry, 2004, enoxaparin	Medical	$14,459 per 1,000 patients with enoxaparin	10 VTE events and 4.4 deaths avoided per 1,000 patients with enoxaparin	$1, 445 per VTE event avoided, and $10,360 per death avoided with enoxaparin	2.7% fewer bleeding events, 0.9% fewer episodes of HIT	Enoxaparin
*Schadlich, 2006, enoxaparin	Medical	Net saving ($46,499) per 1,000 patients with Enoxaparin	N/R	Enoxaparin dominant	7.7 fewer major bleeding episodes with enoxaparin	Enoxaparin
*Deitelzweig, 2008	Medical	Net saving ($339,361) per 1,000 patients with enoxaparin	11 VTE events, three deaths avoided per 1,000 patients with enoxaparin	Enoxaparin dominant	Five major bleeding events, four episodes of HIT avoided per 1,000 patients with enoxaparin	Enoxaparin
Wade, 2008, enoxaparin	Gynecology oncology Surgery	Net saving ($36,197) per 1,000 patients with enoxaparin	Eight DVTs, 18 PE events avoided per 1,000 patients with enoxaparin	Enoxaparin dominant	21 additional major bleeding events per 1,000 patients with enoxaparin	Enoxaparin
*Lloyd, 1997, nadroparin	Orthopedics	Net savings ($192,000) per 1,000 patients with enoxaparin	50 VTE events avoided per 1,000 patients with enoxaparin	Enoxaparin dominant	Not reported	Nadroparin
	General surgery	Net savings ($33,000) per 1,000 patients with enoxaparin	Nine VTE events avoided per 1,000 patients with enoxaparin	Nadroparin dominant	Not reported	Nadroparin
Heerey, 2005, dalteparin	General surgery	$473,000 per 1,000 patients with dalteparin	21 QALYs per 1,000 patients with dalteparin	$20,337/QALY gained with dalteparin	Not reported	Dalteparin
**Low-molecular-weight heparins versus warfarin**						
**Reference**	**Patient population**	**Incremental cost (USD)**	**Incremental effects (VTE avoided or life-years or QALYS gained)**	**ICER (USD/VTE event avoided or life-years or QALYS gained)**	**Bleeding complications**	**Most economically attractive drug**
*O'Brien, 1994, enoxaparin	THR	$133,571 per 1,000 patients with LMWH	Five VTE events, 0.4 deaths avoided per 1,000 patients with LMWH	$26,711 per VTE event avoided, $334,055 per death avoided, $32,158 per life-year gained with LMWH	Not reported	LMWH
*Menzin, 1995, enoxaparin	THR	$69,659 per 1,000 patients with LMWH	20.1 VTE events and 4.3 deaths avoided per 1,000 patients with LMWH	$3,466 per VTE avoided, $16,200 per additional death avoided	Not reported	LMWH
*Hull, 1997, tinzaparin	TKR, THR	Net saving ($52,690) per 1,000 patients with LMWH	60 VTE events avoided per 1,000 patients with LMWH	LMWH dominant	2.2% increase in major bleeding events with LMWH	LMWH
*Hawkins, 1998, enoxaparin	TKR	$126,766 per 1,000 patients with LMWH	145 VTE events avoided per 1,000 patients with LMWH	$874 per VTE event avoided with LMWH	0.3% increased risk of major bleeding event with LMWH	LMWH
*Francis, 1999	THR	Net saving ($153,000) per 1,000 patients treated with LMWH	112 VTE events avoided per 1,000 patients with LMWH	LMWH dominant	62 more patients with bleeding event with LMWH	LMWH
*Botteman, 2002, enoxaparin	THR	$154,000 per 1,000 patients with LMWH	77 DVTs avoided per 1,000 patients, 40 QALYs gained per 1,000 patients with LMWH	$2013 per DVT avoided, $40,169 per death avoided, $4349 per QALY gained with LMWH	Not reported	LMWH
Nerurkar, 2002, enoxaparin	TKR	Net saving ($1, 054,000) per 1,000 patients with LMWH	Seven deaths avoided per 1,000 patients with LMWH	LMWH dominant	Not reported	LMWH
*Caprini, 2002	THR	$110,235 per 1,000 patients with LMWH	5.8 VTE events avoided per 1,000 patients with LMWH	$19,006 per VTE event avoided with LMWH	Not reported	LMWH
**Comparison of low-molecular-weight heparins and other agents**						
**Reference**	**Patient population**	**Incremental cost (USD)**	**Incremental effects (VTE avoided or life-years or QALYS gained)**	**ICER (USD/VTE event avoided or life-years or QALYS gained)**	**Bleeding complications**	**Most economically attractive drug**
Levin, 2001, desirudin versus enoxaparin	THR	$72,000 per 1,000 patients	19.1 life-years gained per 1,000 patients with desirudin	$3,794 per life-year gained	Not reported	Desirudin
*Honorato, 2004, bemiparin versus enoxaparin	TKR	Net savings ($227,000) per 1,000 patients with bemiparin	42 VTE events avoided per 1,000 patients with bemiparin	Bemiparin dominant	Not reported	Bemiparin
*Attanasio, 2001, dermatan sulfate versus UFH 5,000 U, 3 times daily	Surgical cancer	Net saving ($53,000) per 1,000 patients with dermatan sulfate	70 DVTs avoided and 3.1 lives gained per 1,000 patients with dermatan sulfate	Dermatan sulfate dominant	Five additional major bleeding events with dermatan sulfate	Dermatan sulfate
Heerey, 2005, dalteparin 2,500 U versus dalteparin, 5,000 U	Abdominal surgery	$477,000 per 1,000 patients with dalteparin	18 QALYs per 1,000 patients with dalteparin	$24,357/QALY gained with dalteparin	Not reported	Dalteparin 5,000 U
Wade, 2001, tinzaparin versus enoxaparin	Spinal cord injury	$223,259 per 1,000 patients with enoxaparin	Not reported	Not reported	Not reported	Not reported
**Fondaparinux versus enoxaparin**						
**Reference**	**Patient population**	**Incremental cost (USD)**	**Incremental effects (VTE avoided or life-years or QALYS gained)**	**ICER (USD/VTE event avoided or life-years or QALYS gained)**	**Bleeding complications**	**Most economically attractive drug**
*Gordois, 2003	THR, TKR, HFS	$10,000 per 1,000 patients by discharge from hospital with fondaparinux	11 VTE events, 1.9 deaths avoided per 1,000 patients by discharge from hospital with fondaparinux	$1, 077 per VTE event avoided and $5,317 per death avoided with fondaparinux	Not reported	Fondaparinux
*Lundkvist, 2003	THR, TKR, HFS	Net saving ($59,000) per 1,000 patients with fondaparinux	17.9 VTE events, 2.6 deaths avoided per 1,000 patients (average among three conditions) with fondaparinux	Fondaparinux dominant	Not reported	Fondaparinux
Wade, 2003, enoxaparin, 30 mg twice daily enoxaparin, 40 mg once daily	THR	Net savings ($168,382) per 1,000 patients with enoxaparin	Three VTE events per 1,000 patients with enoxaparin	Enoxaparin dominant	27 more bleeding episodes per 1,000 patients with fondaparinux compared with twice-daily enoxaparin Six more bleeding episodes per 1,000 patients with enoxaparin once daily compared with fondaparinux	Enoxaparin twice daily
Annemans, 2004	THR, TKR, HFS	$2,800 per 1,000 patients with fondaparinux	17.7 VTE events per 1,000 patients with fondaparinux	$158 per VTE event avoided; $104 per death avoided with fondaparinux	1.6 more bleeding episodes per 1,000 patients with fondaparinux	Fondaparinux
*Dranitsaris, 2004	THR, TKR, HFS	Net saving ($50,000) per 1,000 patients with fondaparinux	16 VTE avoided per 1,000 patients with fondaparinux	Fondaparinux dominant	10 more major bleeding events per 1,000 patients with fondaparinux	Fondaparinux
Spruill, 2004	TKR (2002 USD)	Net saving ($43,549) per 1,000 patients with fondaparinux	36 VTE events avoided per 1,000 patients with fondaparinux	Fondaparinux dominant	10 more major bleeds and three more minor bleeding events per 1,000 patients with fondaparinux	Fondaparinux
Spruill, 2004	THR (2002 USD)	Net saving ($18,898) per 1,000 patients with fondaparinux	20 VTE events avoided per 1,000 patients with fondaparinux	Fondaparinux Dominant	19 more major bleeding events per 1,000 patients with fondaparinux	Fondaparinux
Wade, 2004	HFS	$21,171 per 1,000 patients with fondaparinux	34 VTE events avoided per 1,000 patients with fondaparinux	$623 per VTE avoided, $32,144 per QALY gained with fondaparinux	Approximately 20% increased bleeding costs for fondaparinux	Fondaparinux
*Sullivan, 2004	THR, TKR, HFS	Net savings ($67,000) per 1,000 patients treated with fondaparinux	3.7 VTE events avoided per 1,000 patients with Fondaparinux	Fondaparinux dominant	Two more bleeding events per 1000 patients with Fondaparinux	Fondaparinux
*Szucs, 2005	THR, TKR, HFS	Net savings ($18,153) per 1,000 patients treated with fondaparinux	8.1 VTE events avoided per 1,000 patients with fondaparinux	Fondaparinux dominant	1.6 more bleeding events per 1,000 patients with fondaparinux	Fondaparinux
*Bjorvatn, 2005	THR, TKR, HFS	$53,553 per 1,000 patients treated with fondaparinux	7.2 VTE events avoided per 1,000 patients with fondaparinux	$753 per VTE avoided, $6,782 per death avoided with fondaparinux	Two more bleeding events per 1,000 patients treated with fondaparinux	Fondaparinux
**Dabigatran versus rivaroxaban and low-molecular-weight heparins**						
Wolowacz, 2009	THR	THR Net savings ($103,050) per 1,000 patients treated with dabigatran	Two VTEs avoided, eight life-years, six QALYs gained per 1,000 patients treated with dabigatran	Dabigatran dominant	Five additional major bleeding events, two episodes HIT avoided per 1,000 patients treated with dabigatran	Dabigatran
	TKR	Net savings ($8,162) per 1,000 patients treated with dabigatran	Four VTEs avoided, 9 life-years, 7 QALYs gained per 1,000 patients treated with dabigatran	Dabigatran dominant	Six additional major bleeding events, two episodes HIT avoided per 1,000 patients treated with dabigatran	
McCullagh, 2009	THR	Net savings ($24,104) per 1,000 patients treated with rivaroxaban	7 Life-years, 10 QALYs gained per 1,000 patients with rivaroxaban	Rivaroxaban dominant	Not reported	Rivaroxaban
	TKR	Net savings ($213,452) per 1,000 patients treated with rivaroxaban	7 Life-years, 12 QALYs gained per 1,000 patients with rivaroxaban	Rivaroxaban dominant		

HFS, hip-fracture surgery; ICER, incremental cost-efficacy ratio; LMWH, low-molecular-weight heparin; THR, total hip replacement; UFH, unfractionated heparin; USD, United States dollars; VTE, venous thromboembolism. *Industry-sponsored study.

#### Unfractionated Heparin versus Low Molecular Weight Heparins

**Figure 2:**
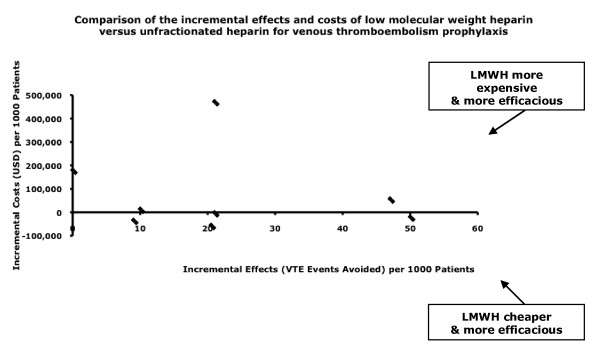
**Comparison of the incremental effects and costs of low-molecular-weight heparin versus unfractionated heparin for venous thromboembolism prophylaxis**.

Among the 12 studies comparing low-molecular-weight heparins with unfractionated heparin among medical and surgical patients, 11 found that low-molecular-weight heparins were more effective (Table 3 and Figure 2). Eight of the 12 studies comparing low-molecular-weight heparins with unfractionated heparin found low-molecular-weight heparins to be the dominant strategy [21,23-25,27,29,30,32,33]. Two studies reported an incremental cost-efficacy ratio of $1,180 and $1,445 per venous thromboembolism event avoided when using enoxaparin [26,29], and two studies found incremental cost-effectiveness ratios of $10,360 per death avoided and $20,337 per quality-adjusted life-year gained with low-molecular-weight heparins. A single study of venous thromboembolism prevention among patients undergoing colorectal cancer surgery found no difference in efficacy, yet costs of low-molecular-weight heparins were greater [28]. Three studies reported increased bleeding risk with low-molecular-weight heparins, and three studies reported lower risk. Sensitivity analyses did not change these results. Of these 12 studies, eight received financial support from the manufacturer of the low-molecular-weight heparins [23-26,28-30,33].

#### Warfarin versus Low-Molecular-Weight Heparins

**Figure 3:**
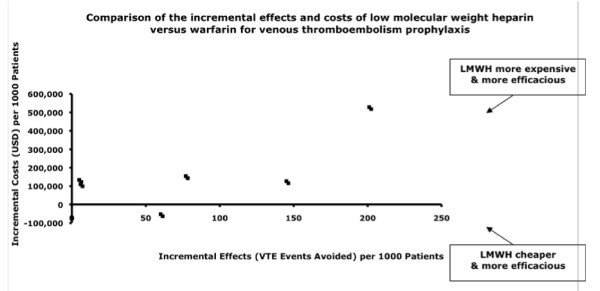
**Comparison of the incremental effects and costs of low-molecular-weight heparin versus warfarin for venous thromboembolism prophylaxis**.

Low-molecular-weight heparins were reported to be economically more attractive than warfarin in all eight studies among surgical patients, with incremental cost-efficacy ratios of $874 to $26,711 per venous thromboembolism event avoided in five of the comparisons [34,35,37,38,40], the dominant strategy in three comparisons [36,39,41]. Long-term outcomes varied widely, with $16,200 to $334,055 per death avoided, $32,158 per life-year and $4,340 per quality-adjusted life-year gained (Table 3 and Figure 3) [34,38]. Sensitivity analyses did not change the results in individual studies. Of these eight studies, seven received pharmaceutical sponsorship [34-38,40,41].

#### Low-Molecular-Weight Heparins versus One another, and Other Comparisons

Within the studies comparing low-molecular-weight heparins with one another and with other anticoagulants among surgical patients, bemiparin and dermatan sulfate were the dominant prophylaxis over enoxaparin [43,44]. Desirudin had an incremental cost-effectiveness ratio of $3,794 per life-year gained, whereas enoxaparin was favored over tinzaparin but was more expensive (Table 3) [45]. Dalteparin, 5,000 units once daily, was more efficacious than dalteparin, 2,500 units, with an incremental cost-effectiveness ratio of $24,357 per quality-adjusted life-year gained [30].

#### Fondaparinux versus Low Molecular Weight Heparins

**Figure 4:**
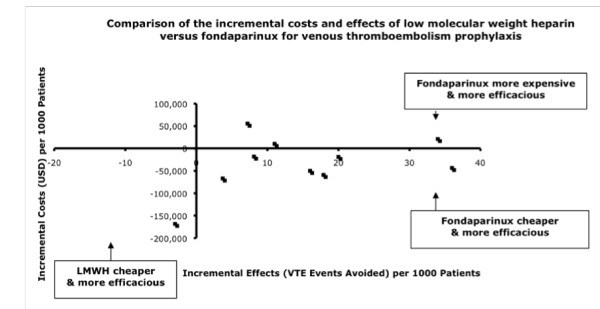
**Comparison of the incremental effects and costs of low-molecular-weight heparin versus fondaparinux for venous thromboembolism prophylaxis**.

Among the 11 studies comparing fondaparinux with enoxaparin, all were conducted in orthopedic surgery patients, and all concluded that fondaparinux was economically attractive. In six, fondaparinux was dominant [47,51-53,55,56], and in one, enoxaparin [48] (Table 3 and Figure 4). In four studies, incremental cost-effectiveness ratios of fondaparinux over enoxaparin were $158 to $1,077 per venous thromboembolism event avoided, $104 to $6,782 per death avoided, and $32,144 per quality-adjusted life-year gained [46,49,53,54]. In eight of 11 studies, fondaparinux was associated with increased bleeding risk. Sensitivity analyses of the various costs did not alter the findings. The manufacturer of fondaparinux provided sponsorship for six of the 11 studies.

#### Dabigatran and Rivaroxaban versus Low Molecular Weight Heparins

Among orthopedic patients, dabigatran, in comparison with enoxaparin, was dominant, with cost savings of $103,050 and $8,162 and six and seven quality-adjusted life-years gained per 1,000 patients with a total hip replacement and total knee replacement, respectively [58]. Comparing rivaroxaban, dabigatran, and enoxaparin among orthopedic surgery patients, rivaroxaban was dominant, with cost savings of $24,104 and $213,452 and 7 life-years gained per 1,000 patients with a total hip replacement and total knee replacement, respectively [57].

#### Sponsorship and Economic Comparisons

When comparing different populations and the different modes of venous thromboembolism prophylaxis, we observed several interesting trends. All studies comparing fondaparinux, rivaroxaban, or dabigatran with enoxaparin were performed in orthopedic patients, and the remainder of the studies in this patient population examined various low-molecular-weight heparins or warfarin. Sixteen of the 25 studies among orthopedic patients were sponsored in some manner by the pharmaceutical industry: six studies favored fondaparinux [46,47,50,54-56]; one, rivaroxaban [57]; one, dabigatran [58]; and the remainder favored low-molecular-weight heparins [25,34-38,40,41,43]. In comparison, five of the eight studies conducted in medical patients compared enoxaparin with placebo [20-24]; two compared unfractionated heparin with enoxaparin [29,33]; and the final one compared enoxaparin with tinzaparin [45]. Five of these eight studies were sponsored by pharmaceutical companies, and all studies favored enoxaparin [20,22-24]. Of the five studies in other surgical populations, three were sponsored by the pharmaceutical industry, and these studies favored unfractionated heparin [28], nadroparin [30], and dermatan sulfate [44].

Of the economic evaluations included in this review, 25 (64%) received funding by manufacturers of one of the comparators. The "new" agent within the comparison was deemed to have a favorable (dominant or attractive incremental cost-effectiveness ratio) outcome in 38 (97.4%) of the 39 economic evaluations (95% CI, 86.5% to 99.9%). Among the 25 studies funded by a pharmaceutical company, the sponsored medication was reported to be economically attractive in 24 (96.0%) (95% CI, 80.0% to 99.9%).

## Discussion

In this systematic review of economic analyses of venous thromboembolism-prevention strategies in hospitalized patients, we found that all of the high-quality studies focused on pharmacologic prophylaxis. Low-molecular-weight heparins were the most commonly studied "new" class of medication and were generally reported to be the dominant or economically attractive strategy in comparison with placebo, unfractionated heparin, or warfarin, among medical and surgical patients. However, among orthopedic patients, fondaparinux was favored over low-molecular-weight heparins. The two newest agents, dabigatran and rivaroxaban, are favored in the two most recent orthopedic surgery evaluations included in this review. Among the various strategies compared, the new agent had a favorable cost-efficacy ratio in 97% of the studies, and most of these studies were sponsored by the manufacturers of the new agent.

Few systematic reviews of economic analyses comparing different modes of venous-thromboembolism prophylaxis in hospitalized patients have been conducted. In 1994, one such review of cost-efficacy analyses of low-molecular-weight heparins, unfractionated heparin, and warfarin in the prevention and treatment of venous thromboembolism in surgical patients, concluded that low-molecular-weight heparin was more efficacious and cost-effective after total hip arthroplasty [34]. Most recently, a trial comparing low-molecular-weight heparin with unfractionated heparin in hospitalized patients found low-molecular-weight heparin to be cost saving compared with unfractionated heparin and that low-molecular-weight heparin was associated with a lower venous thromboembolism readmission rate at 30 and 90 days [59]. Our review includes 37 subsequently published analyses, focusing on both medical and surgical patient populations, and comparing newer pharmacologic agents for venous thromboembolism prophylaxis, such as fondaparinux, dabigatran, and rivaroxaban.

In this review, low-molecular-weight heparins appeared to offer superior prophylaxis efficacy compared with warfarin, unfractionated heparin, and placebo for orthopedic, general surgical, and medical patients. Fondaparinux was found to be economically more attractive for venous thromboembolism prevention compared with heparins because of greater efficacy in surgical and orthopedic patients, but may also be associated with increased bleeding. Among 11 economic analyses comparing enoxaparin with fondaparinux, all found that fondaparinux was economically attractive. More than half of these studies were either directly sponsored by the manufacturers of fondaparinux, or were based on original randomized controlled trials funded by the manufacturer.

Historically, many economic evaluations of new drugs have been sponsored by the drug manufacturer. However, this introduces the potential for bias in model construction and interpretation of the results. In a retrospective analysis of 107 trials in five leading medical journals with regard to outcome and sources of funding, studies sponsored by pharmaceutical companies were much less likely to favor traditional therapy over new drug treatment [60,61]. It is not surprising that new agents are incrementally efficacious; this is the nature of progress in medicine. However, new agents, typically still under patent protection, are virtually always substantially more expensive than comparator drugs. In our systematic review, 25 of the 39 studies were funded by pharmaceutical companies, and, with the exception of a single study [28], each of these found the sponsored drug more economically attractive than the comparator drug. Such consistency in incremental cost-effectiveness among more-expensive drugs is striking. Importantly, we could not detect a consistent bias in outcome between sponsored and nonsponsored evaluations; however, only a minority of evaluations did not receive sponsorship.

Strengths of our review include adherence to rigorous systematic review methods, which consisted of a comprehensive search strategy, broad eligibility criteria, and study selection by two independent adjudicators using *a priori *criteria to minimize selection bias. Economic analyses are susceptible to investigator bias, often due to retrospective decision-model generation and retrospective acquisition of cost-and-effect data. To reduce this risk, we included only economic evaluations that incorporated outcome data from prospective randomized controlled trials. We conducted data abstraction and critical appraisal in duplicate, by using established criteria for assessing economic evaluations. We also addressed the relation of recency to market and for-profit sponsorship in influencing the reporting of economic evaluations. This review also has limitations. Many of the analyses within studies that we included come from a limited number of trials and cost-comparison models. For example, five studies used outcome data from the MEDENOX trial [20,22-24,33]. If most of the data are derived from a limited number of efficacy trials and cost models, similar results are likely to be found across economic evaluations. Although the studies included in this review received high ratings of internal validity, studies varied widely with respect to patient population, time-horizon of therapy, and payer perspective, making generalizability to other health care difficult. In addition, many evaluations rely on radiologic as opposed to clinical venous thromboembolism detection, which may overestimate the real-life clinical consequences of venous thromboembolism. Side effects of thromboprophylaxis may be underestimated, as randomized controlled trials often exclude patients at higher risk of bleeding. Furthermore, trials are generally underpowered to detect differences in rare drug-specific complications such as heparin-induced thrombocytopenia. This may lead to an overestimation of cost-efficacy, as reported in the economic evaluations in this review. Finally, our review included a predominance of orthopedic, general surgery, and medical patients, and thus, our findings may not generalizable to other patient populations.

Among economic analyses in this review, incremental cost-effectiveness ratios were commonly expressed in costs per venous thromboembolism events avoided, and they ranged from $500 to $8,000 per venous thromboembolism event avoided. These ranges are difficult to interpret, as no firmly established willingness-to-pay benchmarks exist for venous thromboembolism prevention. Costs per life-year or quality-adjusted life-year gained were less commonly reported, making economic comparisons of venous thromboembolism-prevention strategies and other interventions in healthcare similarly challenging. Comparing and combining ICERs performed with country-specific costs is challenging, as patient, disease, provider, and health-care system factors may influence transferability. We have not adjusted costs based on country-specific purchasing power parity but have adjusted based on changes in gross domestic product over time, and country-specific exchange rates [62].

An informative economic analysis should include both benefits and harms of interventions and the full associated costs over a relevant time horizon. Full costs of venous thromboembolism prophylaxis were not included in some studies. The common complications of venous thromboembolism prophylaxis include prophylaxis failure, leading to thrombotic events, bleeding, and heparin-induced thrombocytopenia [1]. All 39 studies evaluated in this systematic review accounted for breakthrough thrombotic events; however, only half included bleeding complications (Table 3), and none fully accounted for heparin-induced thrombocytopenia. The omission of these potentially serious complications may considerably affect the cost-comparison data of the individual studies. Most studies ascertained costs retrospectively and from the literature. This is often less complete or less accurate compared with prospective determination alongside a randomized controlled trial. Finally, none of the studies included in this review was prospectively designed before results of the randomized controlled trials were published; accordingly, they may be at risk for subjective decision-tree construction and interpretation.

Few studies have evaluated the cost-effectiveness of mechanical venous thromboembolism-prevention strategies, and none of the existing studies met our eligibility criteria. The UK National Institute for Health and Clinical Excellence have recommended considering graduated compression stockings in most patients, although economic evaluations of mechanical venous thromboembolism prophylaxis have generally been of low quality [63]. The paucity of rigorous evidence about the cost effectiveness of mechanical prophylaxis is striking. However, this may be explained by the fact that manufacturers of mechanical devices are often not required to furnish either effectiveness or cost data to regulatory bodies before their introduction and marketing.

## Conclusion

In this systematic review of economic analyses of venous thromboembolism-prevention strategies in hospitalized patients, we found that low-molecular-weight heparins appear to be the most economically attractive strategy for venous thromboembolism prevention among the majority of medical and surgical patients, whereas fondaparinux is more economically attractive for orthopedic patients. The studies, however, may be at risk of overestimating efficacy and underestimating side effects such as bleeding. Approximately two thirds of all evaluations were directly funded by the manufacturer of the new drug, and such drugs were more likely to be found economically attractive in comparison to other strategies. Limited opportunity for peer-reviewed and independent funding for economic evaluations unfortunately leads to reliance on industry sponsorship in this field. In the future, we recommend that high-quality, prospective, cost-effectiveness analysis be planned alongside the intervention trials and that these be designed, conducted, analyzed, and reported independent of industry sponsors.

## Key messages

• Low-molecular-weight heparins appear to be the most economically attractive strategy for venous thromboembolism prevention among the majority of medical and surgical patients, whereas fondaparinux is more economically attractive for orthopedic patients.

• However, approximately two thirds of all evaluations were directly funded by the manufacturer of the new drug.

• Such drugs were more likely to be found economically attractive in comparison to other strategies.

• Limited opportunity for peer-reviewed and independent funding for economic evaluations may lead to reliance on industry sponsorship and bias in this field.

## Abbreviations

ACP: American College of Physicians; CI: confidence interval; CIHI: Canadian Institute for Health Information; DARE: Database of Abstracts of Reviews of Effects; DRG: diagnosis-related group; HFS: hip-fracture surgery; ICER: incremental cost-efficacy ratio; LMWH: low-molecular-weight heparin; MEDENOX: prophylaxis in medical patients with enoxaparin trial; NICE: National Centre for Clinical Excellence; N/R: not reported; OHIP: Ontario Health Insurance Plan; RCT: randomized controlled trial; THR: total hip replacement; TKR: total knee replacement; UFH: unfractionated heparin; USD: United States dollars; VTE: venous thromboembolism.

## Competing interests

Drs. Pinto and Thirugnanam have no conflicts to disclose. Dr. Fowler's department has received funding for contract research, over the past 5 years from Eli Lilly, Wyeth, Novartis, and Fugisawa. Drs. Cook, Fowler, and Geerts are the primary investigator and collaborators, respectively, on PROTECT (a randomized clinical trial of low-molecular-weight heparin versus unfractionated heparin for thromboprophylaxis in critically ill patients) and DIRECT (a multicenter observational study of low-molecular-weight heparin thromboprophylaxis in critically ill patients with renal impairment). Both are investigator initiated and peer funded, but DIRECT received additional funding from Pfizer, the producers of dalteparin. PROTECT and DIRECT received donations of funding-in-kind for study drug from Pfizer, the makers of dalteparin. The design, conduct, oversight, analysis, interpretation, and write-up of DIRECT was without any influence from Pfizer. PROTECT is ongoing (in the conduct phase); the same conditions hold. Dr. Geerts, over the past 5-year period, has had the following financial relationships that, in a general way, relate to the subject matter discussed in the article or presentation (research grants/support received: Sanofi-Aventis, Pfizer; Bayer Healthcare (pending); consultancies: Astra Zeneca, Bayer Healthcare, Boehringer Ingelheim, Bristol-Myers Squibb, Covidien, Eli Lilly, GlaxoSmithKline, Leo, Pharma, Merck KGaA, Pfizer, Roche, and Sanofi Aventis; honoraria: Astra Zeneca, Calea, Eisai, Oryx Pharma, Pfizer, and Sanofi-Aventis).

## Authors' contributions

Drs. F, T, P, C, and G have made substantial contributions to conception and design, acquisition of data, analysis, and interpretation of data. They drafted the submitted article and revised it critically for important intellectual content. They provided final approval of the version to be published.

## Authors' information

Dr. Fowler is a Career Scientist of the Ontario Ministry of Health and Long-term Care and an incoming Clinician Scientist of Heart and Stroke Foundation of Canada. Dr. Cook holds a Canada Research Chair from the Canadian Institutes of Health Research.

## Supplementary Material

Additional file 1 **Search Strategies and Results**. Click here for file
