# Supplementary material for: Economic analyses of venous thromboembolism prevention strategies in hospitalized patients: a systematic review
Source: Crit Care. 2012 Mar 9;16(2):R43. doi: 10.1186/cc11241 (PMC3964799; doi:10.1186/cc11241)
Supplement: Additional file 1 — Search Strategies and Results. [file cc11241-S1.DOCX]

**APPENDIX 1**

**Ovid MEDLINE(R) 1946 to October week 2 2011**

**Search History Results**

**1 exp Venous Thrombosis/pc [Prevention & Control]/ 6302**

**2 exp Pulmonary Embolism/pc [Prevention & Control] /3935**

**3 exp Venous Thrombosis/ or exp Pulmonary Embolism/63603**

**4 exp Dalteparin/686**

**5 exp Anticoagulants/172110**

**6 exp Heparin/53716**

**7 exp Heparin, Low-Molecular-Weight/8731**

**8 exp Enoxaparin/2196**

**9 exp Nadroparin/401**

**10 exp Vena Cava Filters/1932**

**11 exp Stockings, Compression/692**

**12 exp Intermittent Pneumatic Compression Devices/293**

**13 exp Aspirin/35443**

**14 exp Warfarin/12612**

**15 Fondaparinux/989**

**16 rivaroxaban/353**

**17 dabigatran/412**

**18 exp Venous Foot Pump/6**

**19 exp "Costs and Cost Analysis"/ or exp Cost-Benefit Analysis/146654**

**20 1 or 2 or 3/160809**

**21 4 or 5 or 6 or 7 or 8 or 9 or 10 or 11 or 12 or 13 or 14 or 15 or 16 or 17 or**

**18/205253**

**22 20 and 21/16052**

**23 limit 20 to "economics (sensitivity)"/1349**

***Notes: the bolded MESH terms include following sub-terms**

**Venous thrombosis: deep vein thrombosis**

phlebothrombosis

thrombosis, deep vein

thrombosis, venous

deep venous thrombosis

deep-vein thrombosis

deep-venous thrombosis

**Pulmonary embolus**

pulmonary infarction

pulmonary thromboembolism

thromboembolism, pulmonary

embolism, pulmonary

embolisms, pulmonary

infarction, pulmonary

pulmonary embolisms

pulmonary thromboembolisms

thromboembolisms, pulmonary

**Dalteparin**

Top of Form

tedelparin

dalteparin sodium

fr-860

fragmin

fragmine

kabi-2165

pharmacia brand of dalteparin sodium

pharmacia spain brand of dalteparin sodium

**Heparin**

heparinic acid

alpha-heparin

liquaemin

sodium heparin

**LMWH**

lmwh

low-molecular-weight heparin

low molecular weight heparin

**Enoxparin**

clexane

emt-966

emt-967

enoxaparine

lovenox

pk-10,169

pk-10169

**Compression stockings**

compression stockings

elastic stockings

stockings, elastic

**Pneumatic compression**

compression devices, intermittent pneumatic

pneumatic compression stockings

pneumatic compression hose

pneumatic hose

pneumatic intermittent impulse device

**Aspirin**

acetylsalicylic acid

2-(acetyloxy)benzoic acid

acetysal

acylpyrin

aloxiprimum

colfarit

dispril

easprin

ecotrin

endosprin

magnecyl

micristin

polopirin

polopiryna

solprin

solupsan

zorprin

**Warfarin**

4-hydroxy-3-(3-oxo-1-phenylbutyl)-2h-1-benzopyran-2-one

aldo brand of warfarin sodium

aldocumar

antigen brand of warfarin sodium

apo-warfarin

apotex brand of warfarin sodium

bailly brand of warfarin sodium

boots brand of warfarin sodium

bristol-myers squibb brand of warfarin sodium

coumadin

coumadine

estedi brand of warfarin sodium

gen-warfarin

genpharm brand of warfarin sodium

goldshield brand of warfarin sodium

marevan

tedicumar

warfant

warfarin potassium

warfarin sodium

**Economic analysis**

cost effectiveness

cost-benefit data

benefits and costs

cost benefit

cost benefit analysis

costs and benefits

**EMBASE 1980 to 2011 Week 41**

**Search History Results**

**1 exp Venous Thrombosis/pc [Prevention & Control]/9018**

**2 exp Pulmonary Embolism/pc [Prevention & Control]/4779**

**3 exp Venous Thombosis/ or esp Pulmonary Embolism/101719**

**4 exp Dalteparin/5242**

**5 exp Anticoagulants/389851**

**6 exp Heparin/97764**

**7 exp Heparin, Low-Molecular-Weight/32183**

**8 exp Enoxaparin/12343**

**9 exp Nadroparin/3407**

**10 exp Vena Cava Filters/2428**

**11 exp Stockings, Compression/1377**

**12 exp Intermittent Pneumatic Compression Devices/383**

**13 exp Aspirin/128042**

**14 exp Warfarin/48226**

**15 exp fondaparinux/3682**

**16 exp rivaroxaban/1319**

**17 exp dabigatran/1558**

**18 exp Venous Foot Pump/15**

**19 exp “Costs and Cost Analysis”/ or exp Cost-Benefit Analysis/251841**

**20 1 or 2 or 3/101719**

**21 4 or 5 or 6 or 7 or 8 or 9 or 10 or 11 or 12 or 13 or 14 or 15 or 16 or 17 or**

**18/392084**

**22 20 and 21/34073**

**23 limit 22 to "economics (maximizes sensitivity)"2521**

***Notes: the bolded MESH terms include following sub-terms**

**Venous thrombosis**

Phlebothrombosis

Thrombosis, Venous

Vena Thrombosis

Venous Thrombosis

**Pulmonary embolus**

Chronic Lung Embolism

Embolism, Lung

Lung Embolization

Lung Embolus

Lung Embolus Recurrence

Lung Emboli

Lung Microembolism

Lung Microembolization

Lung Microembolus

Lung Thromboembolism

Microembolus,Lung

Pulmonary Embolism

Pulmonary Embolization

Pulmonary Embolus

Pulmonary Microembolism

Pulmonary Thromboembolism

Thromboembolism, Lung

**Economic analysis**

Cost Effectiveness

Cost Effectiveness Ratio

Cost Efficiency Analysis

Capital Expenditures

Direct Service Costs

Employer Health Costs

Health Care Costs

Health Care Economics and Organizations

Health Care Expenditure

Health Care Sector

Health Expenditures

**All EBM Reviews - Cochrane DSR, ACP Journal Club, DARE, and CCTR, 4^th^ Quarter 2011**

**Search History Results**

**1 exp Venous Thrombosis/pc [Prevention & Control]/925**

**2 exp Pulmonary Embolism/pc [Prevention & Control]/306**

**3 exp Venous Thrombosis/ or exp Pulmonary Embolism/2214**

**4 exp Dalteparin/188**

**5 exp Anticoagulants/7037**

**6 exp Heparin/3602**

**7 exp Heparin, Low-Molecular-Weight/1412**

**8 exp Enoxaparin/526**

**9 exp Nadroparin/86**

**10 exp Vena Cava Filters/20**

**11 exp Stockings, Compression/82**

**12 exp Intermittent Pneumatic Compression Devices/58**

**13 exp Aspirin/3888**

**14 exp Warfarin/933**

**15 Fondaparinux.mp/190**

**16 rivaroxaban.mp/84**

**17 dabigatran.mp/74**

**18 exp Venous Foot Pump/5**

**19 exp "Costs and Cost Analysis"/ or exp Cost-Benefit Analysis/16721**

**20 1 or 2 or 3/2214**

**21 4 or 5 or 6 or 7 or 8 or 9 or 10 or 11 or 12 or 13 or 14 or 15 or 16 or 17 or**

**18/10609**

**22 20 and 21/1341**

**23 limit 22 to “economic (sensitivity)/”1310**

***Notes: the bolded MESH terms include following sub-terms**

**Venous thrombosis**

Phlebothrombosis

Thrombosis, Venous

Vena Thrombosis

Venous Thrombosis

**Pulmonary embolus**

Chronic Lung Embolism

Embolism,Lung

Lung Embolization

Lung Embolus

Lung Embolus Recurrence

Lung Emboly

Lung Microembolism

Lung Microembolization

Lung Microembolus

Lung Thromboembolism

Microembolus,Lung

Pulmonary Embolism

Pulmonary Embolization

Pulmonary Embolus

Pulmonary Microembolism

Pulmonary Thromboembolism

Thromboembolism,Lung

**Economic analysis**

Cost Effectiveness

Cost Effectiveness Ratio

Cost Efficiency Analysis

Capital Expenditures

Direct Service Costs

Employer Health Costs

Health Care Costs

Health Care Economics and Organizations

Health Care Expenditure

Health Care Sector

Health Expenditures
